# 

**Published:** 1882-10

**Authors:** 


### CONTENTS.

### *MISCELLANEOUS.*

PAGE

Management of the Specific Remedy. P. P. Wells, M. D., Brooklyn, New York, . . . . . . . . . . . . . 405

Attention, . . . . . . . . . . . . . . 416

Potentiation makes the Medicine Homœopathic. B. Fincke, M. D., Brooklyn, N. Y., . . . . . . . . . . . 417

Fatal Errors. Ad. Lippe, M. D., Philadelphia, . . . . . . 433

That Resolution of the Institute. T. F. P., . . . . . . . 435

### *CLINICAL BUREAU.*

Hæmorrhoids. A. McNeil, M. D., Jeffersonville, Ind., . . . . . 437

### *BOOK NOTICES.*

Phthisis Pulmonalis, or Tubercular Phthisis. Bœricke & Tafel, . . 440

Cough Symptoms, . . . . . . . . . . . . . 29–36

